# Mechanisms of Network Interactions for Flexible Cortico-Basal Ganglia-Mediated Action Control

**DOI:** 10.1523/ENEURO.0009-21.2021

**Published:** 2021-06-10

**Authors:** Petra Fischer

**Affiliations:** Medical Research Council Brain Network Dynamics Unit, Nuffield Department of Clinical Neurosciences, University of Oxford, OX3 9DU Oxford, United Kingdom

**Keywords:** β, γ, inhibition, movement vigor, phase coupling, STN

## Abstract

In humans, finely tuned γ synchronization (60–90 Hz) rapidly appears at movement onset in a motor control network involving primary motor cortex, the basal ganglia and motor thalamus. Yet the functional consequences of brief movement-related synchronization are still unclear. Distinct synchronization phenomena have also been linked to different forms of motor inhibition, including relaxing antagonist muscles, rapid movement interruption and stabilizing network dynamics for sustained contractions. Here, I will introduce detailed hypotheses about how intrasite and intersite synchronization could interact with firing rate changes in different parts of the network to enable flexible action control. The here proposed cause-and-effect relationships shine a spotlight on potential key mechanisms of cortico-basal ganglia-thalamo-cortical (CBGTC) communication. Confirming or revising these hypotheses will be critical in understanding the neuronal basis of flexible movement initiation, invigoration and inhibition. Ultimately, the study of more complex cognitive phenomena will also become more tractable once we understand the neuronal mechanisms underlying behavioral readouts.

## Significance Statement

Despite tremendous progress in describing how neuronal activity unfolds before and during movements, the mechanisms that trigger the switch from movement preparation to execution, regulate movement vigor and enable movement inhibition remain unknown. Brief synchronization of neural activity within and between cortical sites and the basal ganglia (BG) may be a key factor in controlling these mechanisms. Here, I review the evidence and describe in detail how synchronization may shape firing rates in distinct sites of the cortico-basal ganglia-thalamo-cortical (CBGTC) network to enable flexible action control.

## Introduction

### Distinct motor control operations

One key role of our nervous system is to interpret sensory information to guide movements enabling us to pursue goals shaped by past experiences. Dyskinetic patients are striking examples of how the ability to move when and how we want should not be taken for granted (Mink, 2003).

Despite tremendous progress in describing how neural activity unfolds before and during movements, the mechanisms that allow neural networks to switch from movement preparation to execution remain unknown (Kaufman et al., 2014; Ames et al., 2019). Here, I will argue that the degree of synchrony and relative timing of ensemble activity in motor networks will be a key puzzle piece in understanding how network communication enables the following functions that are essential for flexible behavior:

#### Selective movement initiation

Sensory inputs cause constant streams of spiking activity enabling us to perceive our surroundings, yet sensory-evoked spikes do not cause movements when we intend to sit still. One essential task of an adaptive motor control system thus is to prevent unselective responses to sensory inputs, and instead control movements in response to higher level cognitive commands.

#### Regulation of movement vigor

What mechanisms regulate how fast we move? Considering that unnecessarily vigorous movements would deplete energy stores quickly, an optimally behaving organism needs to regulate movement vigor continually depending on the conditions that yield rewards.

#### Motor inhibition

Motor inhibition can take on various forms, including relaxing antagonist muscles during movement execution or inhibiting actions in response to new sensory information. Rapid interruption or adjustments of ongoing actions are essential, for example, when hunting prey or escaping predators. Finally, easing into stable muscle contractions and maintaining them also requires a process that constrains or inhibits network dynamics from evolving beyond a target range of dynamics.

### The basal ganglia (BG)’s involvement in movement control

The BG are a set of subcortical structures that play a key role in movement invigoration as evidenced by clinical, lesion and stimulation studies (Turner and Desmurget, 2010; Yttri and Dudman, 2016; Park et al., 2020). Discussions about their potential involvement in gating (Klaus et al., 2019) or even selecting actions (Suryanarayana et al., 2019) are ongoing, but particularly the latter is strongly contested (Turner and Desmurget, 2010; Park et al., 2020).

The subthalamic nucleus (STN) and the striatum are the two main input structures of the BG and are innervated to varying degrees by widespread cortical and subcortical areas, resulting in prefrontal, limbic, and sensorimotor inputs that seem to enable interactions between contextual information and motor control operations (Nambu, 2011; Shipp, 2017).

At rest, intact BG output provides tonic uncorrelated inhibition of the thalamus and brainstem structures (Inase et al., 1996; Wilson, 2013; Higgs and Wilson, 2016; Park et al., 2020). Tonic BG output thus is thought to have a suppressive effect on motor output. Such a general motor-suppressive function also seems to play a role in BG-assisted rapid action cancelation (Aron et al., 2016; Chen et al., 2020). Additionally, BG output also appears to be involved in promoting explorative actions if reward attainment is low (Sheth et al., 2011; Humphries et al., 2012), for example, if an animal is hungry and previous actions have not yielded food, BG output may help generate new movement patterns or invigorate old patterns until obtaining a reward. Depending on the motivational state and context, the BG thus seem to control whether movements are held back and how vigorously a movement should be performed.

Classically, the term “action channels” has been widely used when describing hypotheses about BG function, potentially evoking the picture of two actions engaging two physically separate sets of cells. But considering that the same cells can be recruited to perform different actions, such as bringing food to the mouth, displacing a lever or holding a tonic position (Iansek and Porter, 1980; Mink and Thach, 1991a), all involving elbow flexion, this notion may be misleading. Sensorimotor loops in the cortico-BG-thalamo-cortical (CBGTC) network are somatotopically organized (Nambu, 2011; Shipp, 2017), but some cells even respond to both contralateral and ipsilateral movements (Iansek and Porter, 1980), possibly improving bilateral coordination, highlighting that the segregation is blurred. The sheer unlimited combinations of muscle activations to generate new actions can only be controlled by simultaneously activating groups from a finite pool of neurons and adjusting their activation strength. The alternative to having segregated action channels thus are temporary ensembles of spatially dispersed neurons that emerge intermittently to control movements as a result of flexible changes in functional connectivity (Klaus et al., 2019; Carrillo-Reid and Yuste, 2020). In the following, I will thus refer to neurons that are activated during distinct actions as different ensembles.

**Figure 1.**
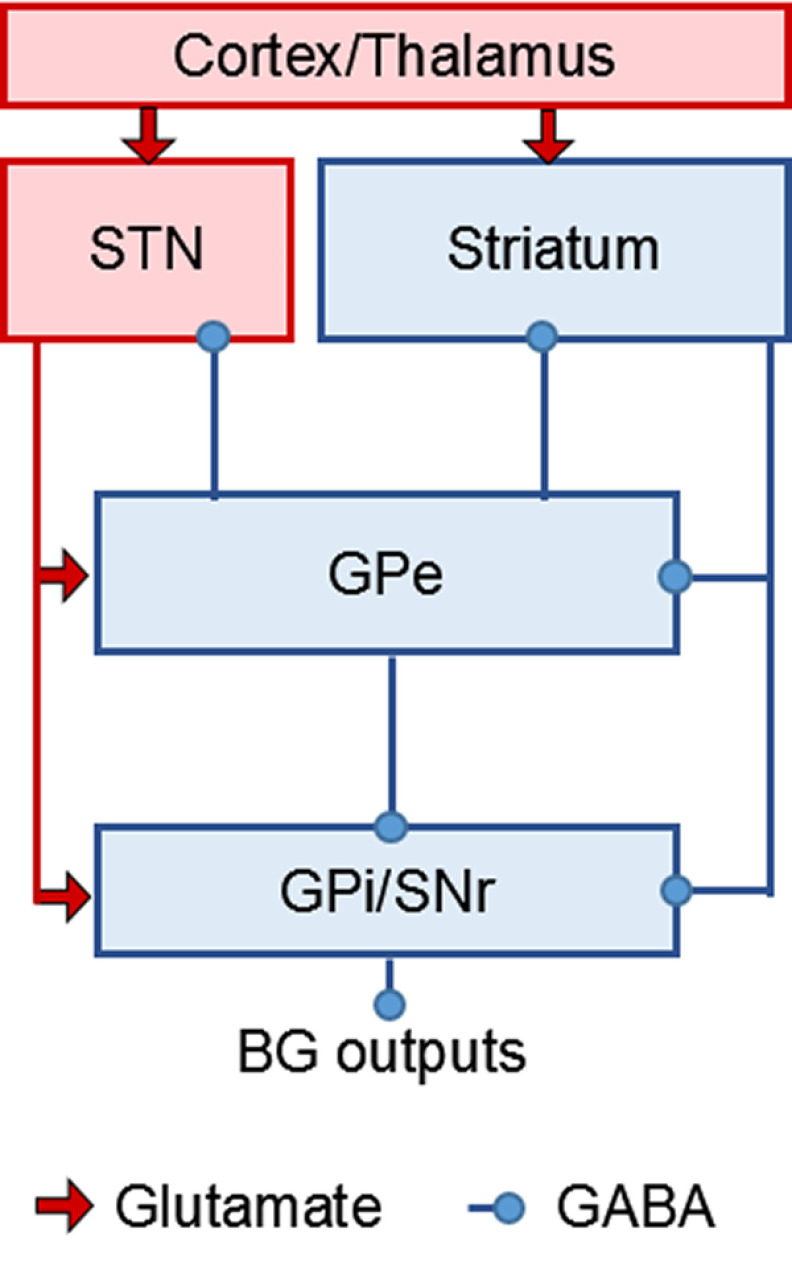
BG architecture. The STN is the only excitatory nucleus within the BG. STN activity excites the GPi and substantia nigra pars reticulata (SNr), the two BG output structures, via direct projections, but also has an indirect inhibitory impact on the GPi via the GPe (Smith et al., 1994; Shink and Smith, 1995; Nambu et al., 2000). The projections between the STN and the GPe, as well as the GPe and the striatum form two recurrent loops potentially promoting oscillations. Excitatory projections are shown in red, inhibitory projections in blue.

The classical box-and-arrow model of the BG posited that a pathway from the striatum→external globus pallidus (GPe)→STN→internal globus pallidus (GPi), also called indirect pathway, should intensify inhibition of the thalamus (Mink, 1996). This is because cortical activation of striatal medium spiny neurons (MSNs) projecting to the GPe and activation of STN neurons projecting to the GPi should lead to increased GPi activity (Fig. 1). Conversely, activation of the direct pathway from the striatum to the GPi is thought to oppose the indirect pathway and result in movement facilitation. However, experimental evidence is inconsistent with a strictly movement-suppressive role of the indirect pathway (Klaus et al., 2019) and has led to speculations that the indirect pathway may also be able to take on a movement-facilitatory role (Calabresi et al., 2014; Mosher et al., 2021). Yet the detailed mechanisms on how this is possible are still unclear.

The STN is a central point of convergence for cortical and subcortical activity (Nambu et al., 1996; Haynes and Haber, 2013; Wilson, 2013) and seems to be involved in both movement invigoration and inhibition (Anzak et al., 2012; Tan et al., 2013; Rae et al., 2015; Wessel et al., 2016; Fischer et al., 2017; Schmidt and Berke, 2017; Lofredi et al., 2021). A recent review highlighted that “a confusing but consistent finding is that most transient [STN] responses can result in both increases and decreases in firing rates […] for both stop and movement responses” (Bonnevie and Zaghloul, 2019). During movement, the majority of movement-responsive STN cells increase firing (Georgopoulos et al., 1983; Pasquereau and Turner, 2017; Pötter-Nerger et al., 2017; Mosher et al., 2021), which quickly subsides when the action is cancelled (Pasquereau and Turner, 2017; Mosher et al., 2021). If STN activity would purely serve to inhibit competing actions (as posited by the classical BG model), it seems counterintuitive that activity of a substantial number of STN cells subsides during action stopping, which is accompanied by broad motor suppression (Wessel et al., 2019).

If the consequences of firing rate changes alone are difficult to understand, what additional features of neural activity could we study? Recently, Park et al. (2020) highlighted in a review on BG function that “it is unclear whether rate models that consider average modulation of output activity […] are sufficient to describe the activity underlying movement execution, and […] BG output may play an even more critical role in modulating precise timing of activity.” In this article, I will thus focus on the aspect of the precise timing of bouts of activity propagating through the CBGTC network and accompanying distinct motor control operations.

### Movement-related synchronization in the CBGTC network

Neurons in the healthy primate BG fire in a temporally relatively uncorrelated fashion (Wichmann et al., 1994; Nini et al., 1995; Bar-Gad et al., 2003) with resting firing rates of ∼50–80 Hz in the GPe and GPi and 15–25 Hz in the STN (Boraud et al., 2002; Wilson, 2013). At movement onset, studies in humans have shown a rapid increase in γ-band synchrony between 60–90 Hz in the contralateral motor cortex (Cheyne and Ferrari, 2013), the STN, the GPi, and the thalamus (Kempf et al., 2009; Anzak et al., 2012; Brücke et al., 2012; Litvak et al., 2012; Singh and Bötzel, 2013; Tan et al., 2013; Lofredi et al., 2018). The spatial site of synchronization is distinct for upper and lower limb movements in line with the known somatotopy in motor cortex (Cheyne et al., 2008) and even in the STN (Tinkhauser et al., 2019).

Combined STN LFP and cortical MEG/EEG recordings further suggest that γ coupling between the STN and cortex is driven by the STN (Litvak et al., 2012; Sharott et al., 2018), indicating that the BG may play a key role in synchronizing neural activity. Simultaneous STN and GPi recordings furthermore showed increased γ phase coupling in Parkinson’s patients in response to dopaminergic medication (Brown et al., 2001; Cassidy et al., 2002), suggesting a potential movement-facilitatory role considering that medication greatly improves their ability to move. In dystonia patients, γ coupling was also observed between the GPi and the thalamus (Kempf et al., 2009).

**Figure 2.**
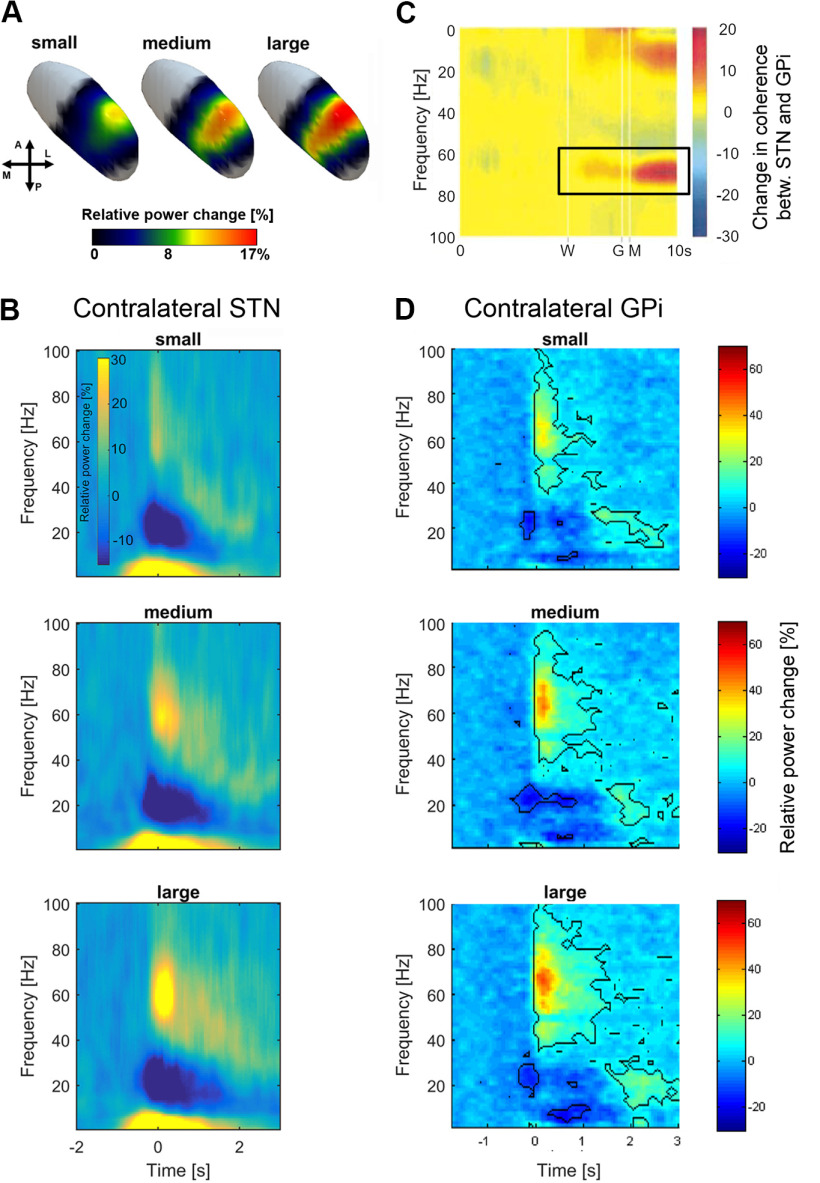
Stronger γ synchronization coincides with increased movement vigor. ***A***, A larger proportion of cells engages in movement-related STN γ synchronization when movements are larger. The task required Parkinson’s patients to perform cued forearm pronation movements. The peak frequency of the movement-related γ increase is similar for small, medium and large movements in the STN (***B***) and in the GPi (***D***)**. *A*** + ***B*** are adapted from Lofredi et al. (2018), and ***D*** is adapted from Brücke et al. (2012). In both studies the peak of γ synchronization seems to follow movement onset. Although not visible here, more subtle changes in synchronization may already occur earlier, similar to the increase in STN-GPi γ coherence as shown in ***C***. ***C***, An early increase in γ coherence (highlighted by the rectangle) was visible between simultaneously recorded STN and GPi LFP activity already after the warning signal (W), which preceded the go signal (G) and movement onset (M) by 2.5 s. This early increase was only apparent on dopaminergic medication in one patient. The sample size was small as simultaneous STN and GPi LFP recordings in humans are very rare. Note that the *y*-axis is vertically flipped compared with ***B*** + ***D***. ***C*** is adapted from Cassidy et al. (2002) by permission of Oxford University Press.

Another striking characteristic of movement-related γ oscillations is that synchronization is stronger when movements are performed more vigorously (i.e., faster, with more force, or bigger; Anzak et al., 2012; Brücke et al., 2012; Singh and Bötzel, 2013; Tan et al., 2013; Lofredi et al., 2018; Fig. 2). Additionally, patients suffering from involuntary movements, such as dystonia and medication-induced dyskinesia also exhibit pronounced cortical γ synchrony and coupling between the STN and motor cortex, raising speculations that γ oscillations may be a causal factor in the generation of dyskinesia (Swann et al., 2016; Miocinovic et al., 2018). This link was recently also confirmed in a rodent model of Parkinson’s disease (Güttler et al., 2021).

Further support for the idea that γ synchronization is closely linked to active movement generation is the observation that movement preparation and passive limb displacements, which both do not involve active muscle contractions, are accompanied by firing rate changes (Crutcher and DeLong, 1984; DeLong et al., 1985; Jaeger et al., 1993; Wichmann et al., 1994), but no pronounced γ synchronization (Cassidy et al., 2002; Liu et al., 2008; Muthukumaraswamy, 2010; Brücke et al., 2012). Considering that γ synchronization specifically peaks at the onset of movements but subsides for the remaining duration of longer movements (Muthukumaraswamy, 2011; Lofredi et al., 2018), it could possibly pose a mechanism that pushes neural dynamics from a preparatory trajectory onto a movement-generating trajectory. What exactly this might entail will be discussed in detail below.

Finally, although most of the studies on human BG activity have been performed in patients with Parkinson’s disease, movement-related γ oscillations in the CBGTC network have also been shown in healthy humans (Cheyne et al., 2008; Muthukumaraswamy, 2010), dystonia patients (Brücke et al., 2008, 2012; Tsang et al., 2012; Singh and Bötzel, 2013), essential tremor patients (Brücke et al., 2013), and healthy rats (Brown et al., 2002; Masimore et al., 2005; von Nicolai et al., 2014; Belić et al., 2016), suggesting that they are a universal phenomenon (Jenkinson et al., 2013; see also Box 1 for more details on the nature of movement-related γ activity).

**Figure 3.**
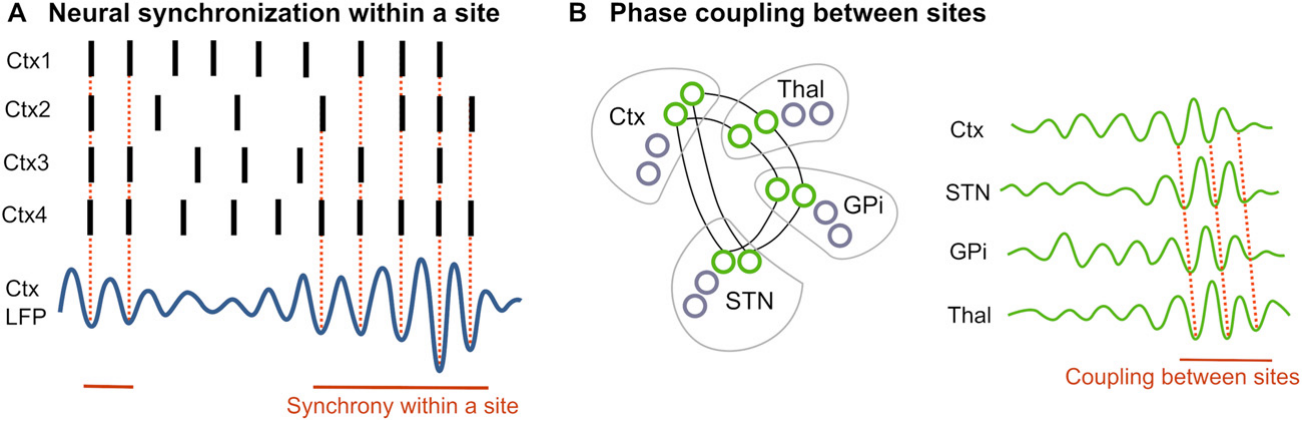
Synchronization within and between sites. ***A***, Synchronization between individual neurons can happen intermittently in bursts of variable lengths within one site. Large-scale local synchronization is reflected as oscillation in the local field potential (LFP). ***B***, I will refer to synchronization between sites as phase coupling. Measures of phase coupling can be obtained by recording LFP activity (or EEG/MEG activity) in two anatomically separate sites and by testing whether the phase of the two oscillatory signals is consistently aligned. In this example, the subcortical sites are driven by cortical activity, with the phases being systematically offset, reflecting conduction delays. Only the green cells representing selected ensembles are synchronized and coupled; the gray cells are not recruited to join the oscillating activity. Directed coherence, Granger causality or dynamic causal modeling (DCM) can be used to make inferences about the directionality of coupling, asking what region is the driver. However, it is important to keep in mind that two recorded sites can be phase-coupled also as a result of being driven by a third site that may have not been recorded (Buzsáki and Schomburg, 2015). Note that phase coupling can but does not need to be accompanied by amplitude coupling. In the example shown in ***B***, the amplitude in subcortical sites increased as the cortical amplitude increased. However, in sites that show strong oscillatory activity at baseline, the EEG/MEG amplitude may decrease when a subset of cells becomes coupled with another site.

Altogether, these observations suggest that rate-based models alone likely are insufficient to understand how CBGTC network activity contributes to movement control. Synchronization of neural activity locally within sites and coupling of synchronous activity between sites, which is commonly assessed with phase coupling metrics, will thus have a central role in this article (Fig. 3). Although here I will focus on the CBGTC network, it is important to note that the BG also directly project to brainstem areas (Mink, 1996; Park et al., 2020), which constitutes another route via which millisecond differences in spike timing may have a substantial effect on muscle control (Sober et al., 2018).

## Which Network Interactions May Coordinate Movement-Related Neural Dynamics?

Changes in firing rates, synchrony and coupling often co-occur, but how do they affect each other? Single- or multi-unit recordings often focus on rate changes, whereas LFP activity recorded from macroelectrodes in patients undergoing deep brain stimulation (DBS) surgery measure fluctuations of population synchrony in the wider vicinity but cannot capture individual spikes. Simultaneous recordings of both LFP and spike activity in multiple sites of the CBGTC network are difficult to obtain in human participants but will be essential to allow investigations of interactions between spike timing, changes in population synchrony and spike rates. In the following, I will discuss four potential mechanisms of network interactions that may be key in facilitating or suppressing movements by manipulating both the timing and rate of spikes.

First, gating of movements may be mediated by a shift in spike timing of cortical cells such that their activity converging in BG sites depolarizes recipient cells more strongly to trigger the firing cascade that causes muscle activation. Second, movement invigoration may depend on coincident activation and temporally clustered inhibition of the relevant ensembles to maximize their impact downstream, generating brief (∼10 ms) synchronized pauses in GPi firing that may promote postinhibitory thalamic activity to boost thalamo-cortical firing rates. Third, incidental co-activation of non-target effector ensembles that are loosely connected with the target-effector ensembles may be avoided by staggering bouts of rhythmic activity, such that incoming non-target-related surround activity would be delayed and thus suppressed by strong local inhibition (potentially occurring at multiple levels of the network). Fourth, rapid suppression of ongoing movements may be enabled by rapid phase or frequency shifts within one of the coupled oscillator networks that are present throughout the CBGTC network to allow an efficient activity reset.

### Mechanism 1: Shifts in spike timing to boost activity

What could mediate the switch from uncorrelated spiking activity at rest to γ-synchronous activity during movement execution? What could be the mechanism that signals “go now” or “go faster,” particularly when no external cues are present?

A considerable fraction of cells that show movement-related increases in firing rates in the CBGTC network tend to fire at higher rates when the movement is performed more vigorously. This has been observed in motor cortex (Cheney and Fetz, 1980; Moran and Schwartz, 1999), the striatum (Kim et al., 2014), the STN (Georgopoulos et al., 1983; Pötter-Nerger et al., 2017), the GP (Georgopoulos et al., 1983; Turner and Anderson, 1997; note that both studies also detected negative correlations between movement amplitude and firing rates in some cells), the substantia nigra (Magarinos-Ascone et al., 1992), and motor thalamus (Gaidica et al., 2018). If the BG indeed control movement vigor, they seem to incorporate a mechanism that regulates local and downstream firing rates.

Box 1: The Fleeting Nature of γ Oscillations

*Gamma synchrony is variable across trials*

A peak in γ synchrony shown in the trial average reflects that the probability of reaching peak synchrony across multiple trials was highest at this point. However, the timing of γ bursts and the degree of synchronization can vary across trials (Lofredi et al., 2018). How meaningful can such synchronization then be? The fact that γ synchronization has consistently been captured in LFP, EEG, and MEG recordings in all CBGTC structures (Kempf et al., 2009; Muthukumaraswamy, 2010; Anzak et al., 2012; Brücke et al., 2012; Litvak et al., 2012; Singh and Bötzel, 2013; Tan et al., 2013; Lofredi et al., 2018) suggests that the actual degree of synchronization between neurons is very large. The process of ramping synchrony up (even if only reaching comparatively weak measurable levels of synchrony in one trial), could thus indeed be causal in pushing the system out of the resting state, activating the neural dynamics resulting in movements. Weak stages of synchronization in spatially distributed neurons that form ensembles may be difficult to detect in LFP recordings from DBS macroelectrodes, but probes with a finer spatial resolution could potentially capture synchronization phenomena that may otherwise be hidden.

*Gamma synchrony quickly disappears after movement onset*

 Finely-tuned ∼60–80 Hz γ oscillations only briefly appear at movement onset and are quickly replaced by slower β oscillations (during relatively stable muscle contractions) or ∼40 Hz oscillations (during more dynamic muscle activation; also called piper rhythm) depending on the movement (Mima et al., 1999; Andrykiewicz et al., 2007; Omlor et al., 2007; Chakarov et al., 2009; Lofredi et al., 2018). These oscillations tend to be coherent with muscle activity (Brown et al., 1998), which can even be enhanced with training (Mendez-Balbuena et al., 2012; von Carlowitz-Ghori et al., 2015). In contrast, finely-tuned 60–90 Hz γ oscillations are only coherent within the CBGTC network, but not with EMG activity (Cheyne, 2013; Jenkinson et al., 2013), suggesting that brief movement-related γ synchronization reflects a central process that drives movement generation or invigoration (Lofredi et al., 2018) independent of proprioceptive feedback.

*Finely-tuned γ captured by different recording methods*

 Whether movement-related γ synchronization clearly stands out in the trial average as a peak with a finely-tuned frequency depends on the recording modality. EEG and MEG sensors measure relatively large spatial sums of cortical population activity, whereas LFPs recorded with DBS electrodes measure local activity at a much finer spatial scale. For recordings from patients with DBS electrodes, the recording contacts need to be close to the γ source considering that movement-related γ synchronization is spatially specific to the dorsolateral STN (Trottenberg et al., 2006; Lofredi et al., 2018). But in general, all three recording methods have been successfully used to capture finely-tuned γ (Muthukumaraswamy, 2011; Brücke et al., 2012; Litvak et al., 2012; Lofredi et al., 2018).

 ECoG contacts over motor cortex instead seem to pick up wide broadband activity (50–300 Hz, or higher) at movement onset (Miller et al., 2007; Fischer et al., 2020), likely resulting from sharp local spiking activity, rendering it more difficult to establish a finely tuned γ peak within the broadband increase. Yet, recently, we showed that even in the presence of superimposed broadband activity, the phase of 60–80 Hz γ oscillations measured with ECoG still carries meaningful information and can provide insights about the spatial localization of cortico-subcortical γ coupling and its relationship to reaction times (Fischer et al., 2020). Hypothesis-driven investigations thus may reveal links between ECoG γ and single-unit activity that have been overlooked so far. Finally, even microelectrode recordings, conventionally capturing spikes, can be used to extract information about local population synchrony after removing individual spikes (Moran and Bar-Gad, 2010; Boroujeni et al., 2020).

**Figure 4.**
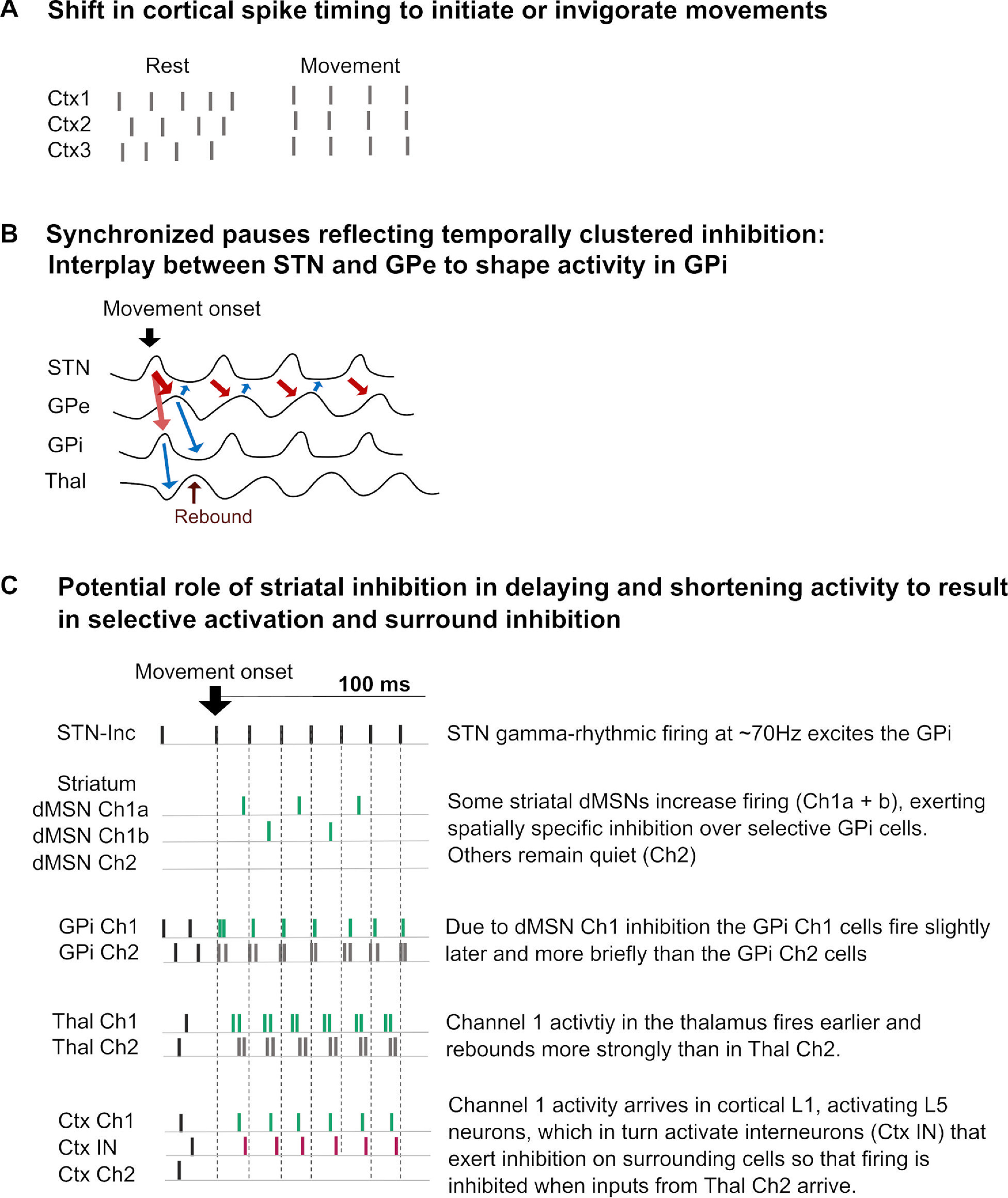
Spike timing-dependent mechanisms of interactions. ***A***, If the spike timing of cortical neurons becomes synchronized, they maximize their impact on downstream cells where their outputs converge, resulting in stronger and faster depolarization (mechanism 1: shift in spike timing). ***B***, γ Oscillations reflecting asymmetric periods of excitation and inhibition could result in prolonged thalamic disinhibition and rebound activity, boosting thalamic firing rates from relatively low baseline firing rates to reach >100 Hz (Goldberg et al., 2013). ***C***, Hypothetical model of surround inhibition through staggered GPi firing. Note that here surround inhibition does not consist of excitation via the direct pathway and inhibition through the indirect pathway as proposed before (Mink, 1996), but instead emerges from temporal offsets in rhythmic activity. During movement onset, a substantial number of STN cells synchronously fire at ∼70 Hz, establishing rhythmic activity in the GPi, while some striatal direct-pathway MSNs also increase and inhibit the GPi more focally (dMSN channel 1). Spikes resulting in movement facilitation are coloured in green. The MSN firing rates at movement onset seem to be substantially lower (∼20 Hz; Alexander, 1987) than those of STN cells, hence GPi target ensembles may not be fully silenced, but instead, their bouts of rhythmic activity, as found in LFP recordings (Brown et al., 2001; Brücke et al., 2012; Tsang et al., 2012; Singh and Bötzel, 2013), may be shorter and delayed (GPi Ch1) relative to the bouts of non-target ensembles that receive no dMSN inhibition (GPi Ch2). Inhibitory GPe activity, which can reach rates of ∼120 Hz during movement execution, could in principle take on a similar role as the dMSN Ch1 cells in reducing and delaying GPi activity (not shown in the schematic). The delayed bouts of GPi Ch1 ensembles would allow thalamic spiking activity in the pauses between successive GPi spikes to occur earlier in Thal Ch1 versus Thal Ch2. The BG-recipient thalamus projects to cortical L1, modulating pyramidal neurons in deeper layers by targeting their dendritic tufts (Garcia-Munoz and Arbuthnott, 2015). The earlier activation of Ctx Ch1 cells may engage a local network of interneurons closing the door to any Thal Ch2 inputs arriving with a delay.

One simple mechanism could involve small shifts in the timing of cortical (and/or thalamic) spikes converging on BG sites. If cortical inputs would arrive in a synchronized, or “bundled” fashion instead of being irregularly dispersed (Fig. 4*A*), they could cause joint activation of thousands of cells, for example, in the STN via the hyperdirect pathway, which could kick off γ oscillations. This mechanism could thus act independently from any apparent changes in cortical firing rates and may potentially require only subtle changes in spike synchronization. Recordings in monkeys have shown that synchrony between motor cortical spikes increased several hundred milliseconds before cortical firing increased when a movement was initiated (Grammont and Riehle, 2003). The synchronization process was linked to movement preparation as it appeared even when the animal only expected a cue to move without later executing the movement. The fact that the cortical synchronization process and the subsequent firing increase were temporally separated suggests that any process that may translate synchronization into increased firing involves additional steps that take place elsewhere. Considering that the STN and the striatum with its expansive cortical inputs are expected to be highly sensitive to changes in spike timing of converging inputs, the BG thus may play an important role in translating synchronization into increased firing rates. Further support for this idea comes from a recent study, in which faster reaction times were preceded by enhanced STN spike-to-cortical γ phase coupling (Fischer et al., 2020) as if coupling slowly built up during movement preparation. During ipsilateral gripping, the timing of STN spikes was clustered around the opposite point of the cycle of cortical γ oscillations, suggesting that movement-related synchronization, which is specific to contralateral movements, depends on the precise timing of STN spikes relative to cortical activity (Fischer et al., 2020). This finding is intriguing, but only two sites of the CBGTC network, the STN and motor cortex, were studied, which makes it impossible to infer the full sequence of network interactions.

Spike integration within short temporal windows also appears to be a key factor in regulating transmission efficacy of GPe cells (Jaeger and Kita, 2011). The recurrent STN-GPe connection (Fig. 1) may have an amplifying role, translating stronger synchrony of inputs into stronger rate changes by recruiting more cells (Fig. 2*A*), potentially enabling a graded regulation of movement vigor through graded synchronization (Anzak et al., 2012; Singh and Bötzel, 2013; Tan et al., 2013; Lofredi et al., 2018). Any such regulation seems to depend also on the motivational state signaled by the striatum (Niv et al., 2007; Liljeholm and O’Doherty, 2012; Crego et al., 2020) and a sense of urgency, which also affects decision times (Carland et al., 2019). Dopamine levels seem to play a key role in invigorating and potentially even permitting movements (Klaus et al., 2019), and exert complex effects not only on the striatum but on all BG nuclei (Mallet et al., 2019). Studies in patients with Parkinson’s have repeatedly shown that γ synchronization is weaker after dopamine withdrawal (Brown et al., 2001; Cassidy et al., 2002; Williams et al., 2002; Alegre et al., 2005; Lofredi et al., 2018), but currently it is unclear how subsecond fluctuations in dopaminergic activity interact with the degree of neural synchronization within the BG and between cortico-BG recording sites. It is also unknown to which extent striatal activity may contribute to the process of generating γ synchronization. Notably, strong external cues, such as loud sounds, can compensate for dopamine depletion at least to some extent and result in faster movements as well as stronger STN γ synchronization (Anzak et al., 2012), indicating that sensory activity can boost subcortical γ activity. External cues can also help patients with Parkinson’s to initiate and maintain walking movements (Ginis et al., 2018). Related to this, a recent study in rodents showed that auditory go cues triggered prepared movements by activating midbrain reticular and pedunculopontine nuclei, which drove the thalamus to rapidly reorganize motor cortical preparatory activity and kick off movement dynamics (Inagaki et al., 2020). These midbrain structures thus may be key for executing externally cued movements. They are also reciprocally connected with the BG (Martinez-Gonzalez et al., 2011).

As an alternative to the hypothesis that the BG receive temporally structured inputs, the BG may simply receive higher rates of uncorrelated cortical and thalamic inputs that trigger γ oscillations within internal BG loops purely because of anatomic constraints. Interestingly, the peak frequency of movement-related γ oscillations tends to be similar regardless of the movement vigor (Fig. 2*B*,*D*), which suggests that the duration of the windows of brief depolarization and hyperpolarization remains relatively stable. If the rate of excitatory inputs to the BG was markedly higher for large versus small movements, then the peak frequency of the subcortical γ oscillations could potentially reflect this change, considering that, for example, visual cortical γ oscillations have a higher peak frequency when the stimulus-induced excitatory drive is stronger (Ray and Maunsell, 2010; Orekhova et al., 2020). However, relatively stable γ peak frequencies may potentially also simply originate from intrinsic properties of STN and GPe cells.

Even if movement-related γ synchronization were to emerge purely because of anatomic constraints, γ-rhythmic activity may still entail functional consequences that will be discussed in the next sections. In comparison to slower oscillations, the relatively fast fluctuations between 60–80 Hz seem more suitable for boosting firing rates, considering that longer periods of relative inhibition may limit rates. To shed light on the limits of different oscillation speeds in shaping firing rates, biologically constrained computational models of the CBGTC network could be used to study interactions between inputs of different frequencies and resulting changes in rates and synchrony.

To sum up this section, I have proposed that changes in spike patterns and correlations within cortical but also between cortico-subcortical sites might be the first measurable phenomenon preceding movement initiation, building up until a tipping point is reached to trigger a cascade of firing rate changes that kicks off the movement. Alternatively, if the role of the BG is limited to regulating movement vigor without actually gating initiation, γ synchronization may still play a mechanistic role in shaping actions as outlined below.

### Mechanism 2: Brief synchronized pauses reflecting temporally clustered inhibition

Each γ cycle reflects membrane potential fluctuations capturing successive periods of depolarization and hyperpolarization. Cortical γ oscillations originating in E:I circuits have been shown to entail brief periods of excitation (∼3–4 ms) followed by prolonged periods of inhibition (∼10 ms; Hasenstaub et al., 2005; Okun and Lampl, 2008; Buzsáki and Wang, 2012; Fries, 2015). The asymmetry arises from a fast succession of principal cells that rapidly activate local inhibitory interneurons, which exert feedback inhibition that slowly subsides, allowing another volley of principal cell activation (Buzsáki and Wang, 2012; Fries, 2015). Whether similar asymmetries exist in the cycles of excitation and inhibition underlying BG γ oscillations is currently unknown. Characterizing such asymmetries would be highly informative, considering that brief synchronized pauses of GPi activity could help boost thalamic firing rates by repeatedly removing GPi-mediated inhibition for the duration of ∼10 ms (visualized in Fig. 4*B*). Assuming a firing rate of 70 Hz, highly rhythmic firing would result in interspike intervals of 14 ms.

What evidence supports the idea that synchronized and potentially prolonged pauses play a role in motor control? Studies in songbirds have demonstrated “paradoxical co-activation” of connected pallidal and thalamic neurons during singing: simultaneous increases in firing rates occurred in both neurons despite the inhibitory nature of the pallidal projection. Pallidal spikes first ensued in powerful but very brief inhibition, silencing thalamic firing for 5 ms, but reliably triggered spiking thereafter, resulting in precisely time-locked activity (Goldberg et al., 2013).

In mammals, individual thalamic neurons receive inputs from multiple pallidal cells, including even projections from the contralateral GPi (Hazrati and Parent, 1991a). Hence, relieving thalamic neurons from BG output-mediated inhibition may depend on coordinated pausing of a large number of GPi cells. Currently, LFP recordings in dystonia and Parkinson’s patients have only provided indirect evidence for this idea. Such recordings consistently showed an increase in movement-related 60–90 Hz GPi synchronization, suggesting that GPi activity becomes more γ-rhythmic (Cassidy et al., 2002; Brücke et al., 2008, 2012; Liu et al., 2008; Kempf et al., 2009; Tsang et al., 2012; Singh and Bötzel, 2013).

One caveat of these studies is that these patients were selected to receive DBS surgery because of motor symptoms resulting from pathologic changes in BG activity. In healthy non-human primates, recent spike-to-spike coupling analyses showed no clear evidence of synchronization (Schwab et al., 2020; Wongmassang et al., 2020), but this does not rule out spike-to-γ phase coupling, which was not directly investigated. Spike-to-γ phase coupling assesses the spike timing relative to population activity, and the advantage of the population average is that it filters out the spike timing variability of individual cells. Moreover, the authors of one of the studies also performed computational simulations, which suggested that GPi→thalamus communication strongly depends on the strength of synchronization between GPi spikes (Schwab et al., 2020).

Two additional points indicate that the movement-related subcortical γ synchronization observed in patients is not merely pathologic. First, after dopamine depletion, BG activity becomes more synchronized for oscillations below 30 Hz in both humans and non-human primates, but oscillations in the γ range tend to be attenuated (Brown et al., 2001; Williams et al., 2002; Deffains et al., 2016). Second, although we cannot access subcortical LFPs in healthy humans, we can still observe movement-related γ synchronization in motor cortex (Cheyne and Ferrari, 2013), which is reciprocally connected with the BG-recipient thalamus (Bosch-Bouju et al., 2013).

In spike recordings of the non-human primate GPi, the number of cells that increases firing during movement outnumbers those that decrease (Anderson and Horak, 1985; Nambu et al., 1990; Mink and Thach, 1991a; Turner and Anderson, 1997, 2005; Schwab et al., 2020). The fact that the majority of cells in the thalamus also increase firing despite the inhibitory GPi→Thal connection is still a conundrum and difficult to reconcile with classical models of BG functions (Schwab et al., 2020). Notably, mean interspike intervals seem to remain above 10 ms even when GPi firing increases to 120 Hz at movement onset (Schwab et al., 2020; see their Supporting Fig. S4). If GPi firing is more synchronized, then the ensuing pauses of activity also occur together, potentially allowing more time for thalamic cells to fire than when GPi activity is lower but asynchronous. Pauses following activation could even trigger thalamic rebound activity (Person and Perkel, 2007; Bosch-Bouju et al., 2013; Kim et al., 2017). Hence, stronger GPi firing including synchronous pauses could thus not only allow cortico-thalamic excitation but potentially even actively boost thalamic firing.

Paying special attention to synchronized pauses may also be helpful considering that single neurons tend to skip cycles even when participating in oscillating population activity (Hasenstaub et al., 2005). The timing of joint silence could thus serve as a reliable sign of temporally clustered inhibition. Analysing spikes *and* pauses will also be important when trying to understand the recurrent interactions between the thalamus and the GABAergic thalamic reticular nucleus (TRN), which also receives direct inputs from the GPe (Hazrati and Parent, 1991b; Mastro et al., 2014). The TRN shows movement-related increases in activity (Saga et al., 2017), but currently it is not known whether the activity is γ-rhythmic. It seems likely, considering that neurons of both the TRN and the thalamus can switch between tonic and bursting firing modes and the reciprocal connections between the TRN and the thalamus appear to promote reverberating oscillations (Halassa and Acsády, 2016). Moreover, TRN bursts can also facilitate postinhibitory spiking (Kim et al., 2017). The TRN is thought to regulate thalamic firing probability more broadly, while pauses of GPi activity were postulated to trigger spatially relatively focal entrainment of thalamic spikes (Halassa and Acsády, 2016). Relative shifts in pauses of GPi and TRN activity thus may be another factor in shaping movement control.

### Mechanism 3: Staggered activity to prevent co-activation of non-selected ensembles

One corollary of boosting firing rates to invigorate movements may be an increased risk to coincidentally activate connected ensembles that are to remain silent. If cells within the target ensembles fire at high rates, then at various stages of the network some level of depolarization likely also spreads to cells that are anatomically connected but target non-selected muscle groups. To prevent them from firing, they may need to be inhibited more strongly.

The BG indeed seem to have the potential to regulate muscle co-activations considering that muscle rigidity is a hallmark symptom of Parkinson’s disease and MPTP lesions, which are both accompanied by altered BG firing patterns and excessive synchronization between 10–30 Hz (Wichmann, 2019). Muscle co-contractions can also occur after inhibiting BG output activity by injecting muscimol into the GPi (Mink and Thach, 1991b; Inase et al., 1996).

Theories about a role of the BG in surround inhibition have existed for many years, postulating that the movement-related increase in GPi activity caused by indirect pathway activity fulfils the purpose of broadly inhibiting competing motor programs, while direct striatal projections cause focal GPi inhibition and thus selective movement facilitation (Mink and Thach, 1993; Mink, 1996). But considering the presence of γ oscillations in the GPi and thalamus at movement onset (Brücke et al., 2008, 2012, 2013; Kempf et al., 2009) and the correlation between changes in firing patterns and motor impairments (Neumann and Kühn, 2017), surround inhibition may depend crucially on the relative spike timing of cells engaging in rhythmic firing.

Where multiple inputs, some excitatory, others inhibitory, converge onto a cell, the relative timing of these inputs determines whether and when the cell fires. I propose a model, in which the STN (together with the GPe) sets a rhythm that strongly shapes GPi activity, which is modulated via inhibitory direct-pathway striatal MSNs (dMSNs; Hazrati and Parent, 1992). Instead of shutting down the selected GPi ensembles fully to disinhibit the thalamus, dMSN activation may simply delay spiking within each γ cycle, so that the resulting GPi pauses can trigger earlier thalamic activation entailing local inhibitory mechanisms at subsequent stages.

Figure 4*C* shows how delaying activity at the level of the GPi through dMSN inhibition may enable inhibition of non-selected ensembles surrounding the target ensembles at the motor cortical stage. In this hypothetical model, selective activation of dMSN channel 1 cells (targeting the intended muscle activation) shortens bouts of firing of the focally targeted GPi ensembles (GPi channel 1) but not of the surrounding ones (GPi channel 2). The GPi channel 1 ensembles that facilitate the selected action are thus not completely silenced by striatal dMSN channel 1 cells, but their spiking is only delayed and reduced. The shorter GPi channel 1 bouts would then result in earlier thalamic disinhibition (Thal channel 1), which triggers earlier cortical activation (Ctx channel 1) that in turn triggers local interneurons (Ctx IN). These interneurons then cut off any thalamic inputs arriving during periods of strong local inhibition (Thal channel 2→Ctx channel 2), effectively stopping non-selected ensembles from firing with the activated ones. In this example, the selected ensembles at the level of the thalamus simply fired earlier in each γ cycle than the non-selected ones. Note that this schematic does not show that some STN cells also exhibit a firing decrease, which could also add to a delay or reduced firing in the GPi. Additionally, selectively increased GPe firing may also have a similar effect. The fact that not only the GPi but also the GPe contains cells that can be negatively or positively correlated with movement amplitude for both movement-related response types (showing either an increase or decrease in firing; see Turner and Anderson, 1997; their Fig. 14) suggests that the dMSN pathway is not the only pathway via which selective thalamic disinhibition takes place.

In motor cortex, surround inhibition indeed seems to aid the selective execution of movements (Beck and Hallett, 2011), and reports of a disrupted mechanism in preclinical Parkinson’s disease suggest it depends on BG signals (Shin et al., 2007). However, currently it is unclear how exactly BG signals contribute, and to which extent surround inhibition is coordinated locally within cortex (Beck and Hallett, 2011). It is also unclear whether the mechanisms that contribute to relaxing antagonist muscles, which seem to break down when rigidity emerges as symptom, overlap with the mechanisms that prevent random unintended movements, which can be observed when patients experience dyskinesia. Note that during movement, a substantial proportion of motor cortical principal cells also decrease activity (27% of corticospinal neurons in one study; Ebbesen and Brecht, 2017; Soteropoulos, 2018), which may be mediated by lateral inhibition.

Finally, at the level of the thalamus, for example, the TRN could take on a similar role to those of cortical interneurons. Hence, timing-based mechanisms to suppress co-activation of non-selected ensembles as laid out in Figure 4*C*, may be relevant at several network levels.

The considerations outlined here do not cover all possible interactions but serve to highlight that investigating the within-cycle organization and relative shifts of activity in distinct ensembles may be essential to advance our understanding of selective movement facilitation and suppression. Why would shifts in spike timing and local inhibitory mechanisms be better suited for selectively facilitating movements than non-rhythmic changes in activity? The former may simply emerge from the network architecture and may require less dramatic deviations from resting state dynamics than the latter.

The idea that propagation of spiking activity can be regulated via small shifts in oscillatory frequencies is also supported by the following observation of corticocortical information transmission: selective allocation of visuospatial attention has been linked to accelerated γ oscillations in ensembles activated by the attended stimulus (Bosman et al., 2012). Conversely, information about competing stimuli is thought to be relatively suppressed as spikes encoding unattended stimuli arrive within periods of local inhibition (Bosman et al., 2012). Whether similar information routing principles can also be found in the CBGTC network has been largely unexplored, despite growing evidence for the idea that γ oscillations can render neural communication effective, precise, and selective (Fries, 2015; Rohenkohl et al., 2018).

### Mechanism 4: Phase or frequency shifts to cancel or change movements

Another remarkable feat of motor network activity is the flexibility to switch population dynamics midway through a movement on an unexpected sensory cue to rapidly cancel or change an action (Ames et al., 2019). To enable fast action stopping, the STN appears to be rapidly activated by two cortical areas, the presupplementary motor area (pre-SMA) and right inferior frontal gyrus (IFG; Aron et al., 2014, 2016; Rae et al., 2015; Chen et al., 2020; Lofredi et al., 2021). Here, I will describe how shifts in spike timing could play their part in this process.

STN LFP and EEG recordings during rapid stopping of an ongoing movement in response to an unpredictable sound showed that local STN γ rapidly increased while STN-to-motor cortical γ coupling dropped (Fischer et al., 2017). The local γ increase seems counterintuitive at first, as STN γ also increases during movement initiation, but the simultaneous drop in STN-to-motor cortical coupling points toward a gating mechanism that rapidly cancels propagation of γ activity through the network.

**Figure 5.**
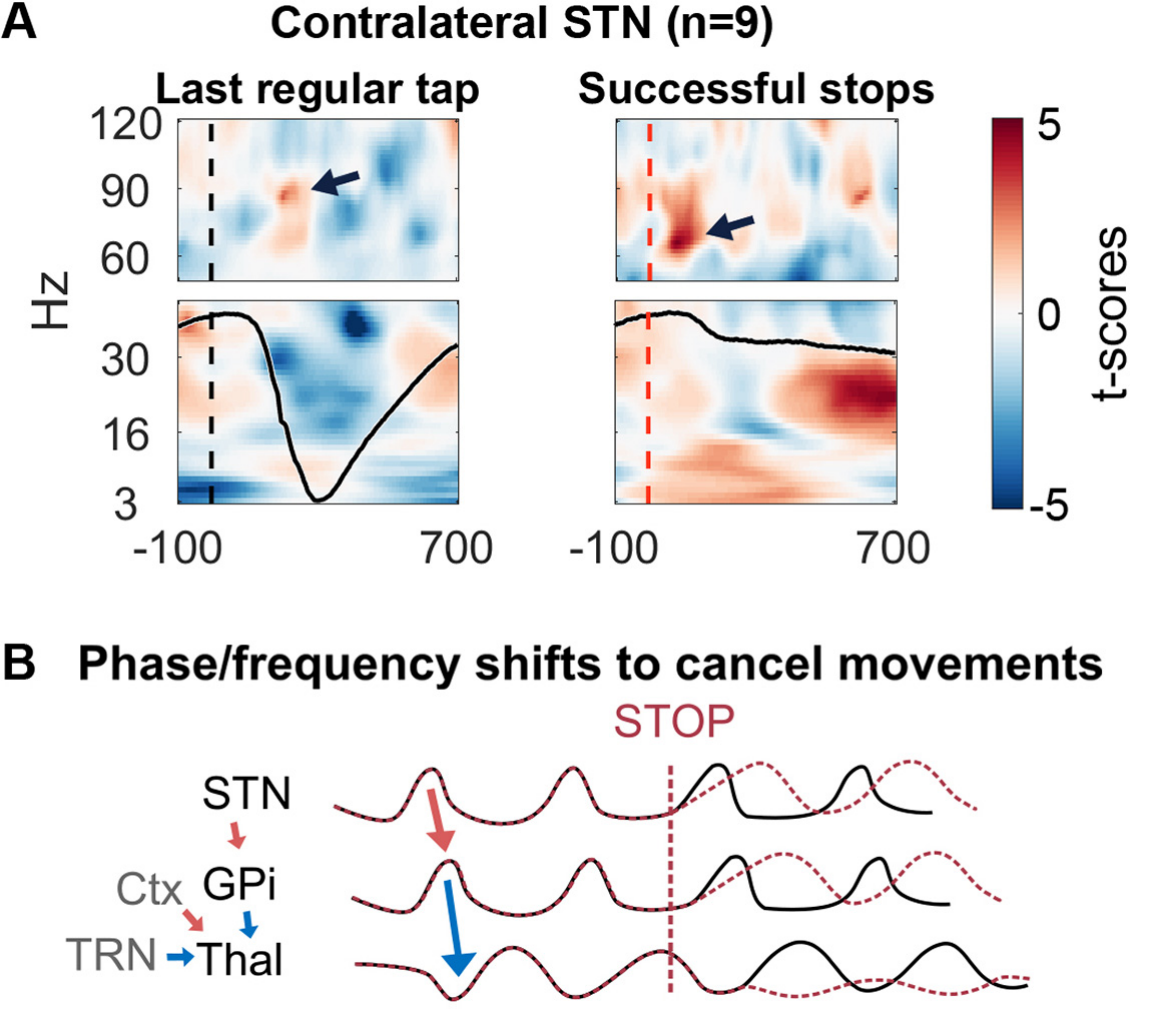
Stop-related activity. ***A***, STN power recorded during finger tapping (left) and successful stopping (right). The γ increase observed during the last regular tapping movement (= the final tap before the stop signal) peaked at around 90 Hz (shown by the arrow), while the γ increase during successful stopping peaked between 60–70 Hz. Peaks at 90 and 65 Hz correspond to γ cycles lasting 11 and 15 ms, respectively (including excitation and inhibition). A lower peak frequency could thus indicate slightly prolonged STN spiking within each cycle. The black curve in the lower panels denotes the finger movement (Left: The finger was first elevated, then it moved down to touch the table at around 300 ms and moved up again. Right: After the auditory stop signal, the downward movement stopped quickly, just after the γ increase.) ***A*** is adapted from Fischer et al. (2017). ***B***, Proposed mechanism: increased drive to the STN after the stop signal may result in prolonged excitation and longer γ cycles (red dashed lines) compared with movement-related activity (black lines, also see Fig. 4*B*). The shifted rhythm is passed on to the GPi. GPi inhibition, cortical excitation and TRN inhibition converge in the thalamus, where they may cancel each other out.

When activity that promotes a movement or triggers movement-promoting dynamics is γ-rhythmic, then these commands could potentially be flexibly and efficiently cancelled by well-timed brief bursts of inhibition (Fig. 5*B*). Specifically, small phase shifts within one part of a network of coupled oscillators may already be sufficient for excitatory and inhibitory activity to “collide” with each other and cancel the former out.

At what level of the network might that occur? Figure 4*C* shows how γ synchronization could structure activity of selected and non-selected action channels during movement initiation. Once the initiation process has started, cortico-thalamic activity becomes γ-rhythmic. Thalamic neurons then receive both γ-rhythmic excitatory cortical and inhibitory BG inputs. Depending on the relative timing, activity in the cortico-thalamic and BG-thalamic oscillators may have an amplifying effect on movement speed. But if they are suddenly pushed out of sync, the inhibitory volleys from the BG may cause sudden activity cancelation and movement cessation.

Rapid phase and frequency shifts of BG outputs could be achieved either by strong cortical inputs to the STN (Rae et al., 2015; Chen et al., 2020) or through γ-rhythmic cortical inputs that shift STN γ accordingly. Frequency-shifting and/or phase-shifting oscillatory activity may be a powerful mechanism to rapidly cancel or re-route activity without spending vastly more spikes. The stop-related STN γ increase seemed to have a lower peak frequency than the movement-related γ increase observed before the stop signal (Fig. 5*A*), providing some support for this idea. The lower γ frequency suggests a longer duty cycle, possibly reflecting prolonged STN spiking within each γ cycle, which could promote prolonged GPi activation within each cycle and more powerful thalamic inhibition. Currently it is unclear whether movement-related and stop-related STN γ synchronization involves distinct sets of cells with different connectivity profiles. Considering that stop-related increases of STN firing activity seem located more ventrally compared with movement-related activity (Pasquereau and Turner, 2017; Chen et al., 2020), these ventral cells may be the ones that trigger the γ shift by engaging the GPe (see Box 2 for details on stop-related activity in the GPe).

Note that STN γ activity may potentially only appear during stopping or switching of an *ongoing* movement, considering that conventional stopping paradigms, which require abortion of a planned button press, have rarely reported γ synchronization and mostly focused on slower β oscillations [13–30 Hz; Aron et al., 2016; Wessel, 2020; and references therein; exceptions are broadband γ increases at the cortical level (Swann et al., 2012; Fonken et al., 2016) or STN γ changes that were temporally strongly smoothed (Ray et al., 2012)]. However, recent studies found that β oscillations appeared only after the stopping process and are thus unlikely part of the causal chain of cortico-STN-mediated stopping (Chen et al., 2020; Mosher et al., 2021). Rather it seems as if pre-SMA and IFG work together to evoke an STN response (Rae et al., 2015; Chen et al., 2020) triggering the switch in the neural dynamics to cancel a movement, which may then be followed by increased β synchronization reflecting stabilization of network dynamics and thus the motor state.

### Understanding the role of slower oscillations

Bursts of CBGTC β oscillations have not only been hypothesized to have a role in stopping but also in sensorimotor integration, updating motor predictions, preserving the current motor state and clearing out previous motor plans (Schmidt et al., 2019). In the context of corticocortical information processing, α (8–12 Hz) and β oscillations, have also been associated with top-down control of working memory, allocation of attention and pattern categorization (Fries, 2015; Miller et al., 2018; Wutz et al., 2018).

Here, I would like to propose that instead of linking the phenomenon of β oscillations to labels describing distinct behavioral functions, their functional relevance may be better understood by investigating their role in shaping concurrent and subsequent network dynamics.

In a continuous force control task, STN β synchronization was positively correlated with slowing of a force adjustment as well as more accurate completion (Fischer et al., 2019), suggesting that β synchronization may be beneficial for ending a dynamic adjustment in a controlled fashion. Also in motor cortex, sustained isometric contractions tend to be accompanied by increased β oscillations and cortico-muscular β coherence (Brown, 2000; Omlor et al., 2007). Local β synchronization and long-range β coupling thus may engage distributed cells to shape activity such that the neural dynamics remain within a certain range and do not cross a threshold that would kick off movement dynamics. This fits with the observation that β synchronization in motor cortex and the STN emerges independently of changes in firing rates (Rule et al., 2017; Cagnan et al., 2019; Confais et al., 2020).

The idea that β synchronization may be relevant for reining in evolving activity that would have led to changes in motor output is also in line with β oscillations appearing when a movement plan is interrupted. An extreme form of stabilization can again be seen in Parkinson’s disease, where excessive β synchrony as a result of dopamine depletion is strongly linked to rigidity and bradykinesia, pathologic overstabilization of motor activity (Little and Brown, 2014; Neumann and Kühn, 2017; Wichmann, 2019).

Linking β synchronization merely to functional consequences that are time-limited to the brief periods of synchronization is difficult to reconcile with the observed trial-to-trial variability of the precise timing of intermittent bursts of β synchronization relative to movement initiation (Feingold et al., 2015; Torrecillos et al., 2018). My key prediction instead is that the effect of intermittent β synchronization on motor network dynamics is longer lasting. If this hypothesis is true, then future studies may confirm that a minimum duration of β-free activity is needed in motor cortices and/or subcortical structures to kick off movement initiation. Only recently, thalamo-cortical recordings in essential tremor patients showed that coupling between the phase of thalamic <30 Hz activity and the amplitude of cortical high-frequency activity consistently dropped before a hand movement was made, as if it reflected movement gating by releasing the cortical high-frequency activity from the thalamic <30 Hz oscillations (Opri et al., 2019). Understanding how the impact of β bursts on prolonged network dynamics differs depending on whether they appear in the BG, the thalamus or motor cortex, may help us pin down the conditions that permit or even promote the onset of movement-related neural dynamics.

Finally, the probability of β bursts is known to increase again after movement completion, particularly if the movement resulted in the expected outcome (Tan et al., 2014; Torrecillos et al., 2015). It suggests that β oscillations may also play a role in maintaining current sensorimotor predictions either by maintaining the current network dynamics or by preventing updating of synaptic weights.

In summary, to advance our understanding of the network interactions leading to movement generation we may need to study not only concomitant but also longer lasting effects of β synchronization on network dynamics.

Box 2: Outstanding Questions

**• Are movement-related γ oscillations triggered by loops within the BG in response to an increased temporally unstructured (asynchronous) cortical drive or are they caused by synchronized inputs?**

**• Do inputs to the BG have different temporal structures depending on whether their purpose is to (1) invigorate actions, (2) cancel an ongoing action, or (3) stabilize movement dynamics, for example, during sustained contractions or when remaining still when an action is cancelled before it was initiated?**

**• Which types of cells engage in movement-related γ synchrony?**

 Different cells throughout the BG can exhibit action-specific (specific to an effector and the movement direction) or non-specific increases or decreases in firing rates or multi-phasic responses. It is currently unclear to which extent these subsets are coupled to LFP γ rhythms at movement onset and if they are all locked to the same phase. To understand interactions between different ensembles and different sites, it will be key to quantify the coupling strength and the preferred phase relative to local synchronization captured by the LFP. It will also be important to test to which extent cells that show no changes in firing rates contribute to γ synchronization.

**• Can we detect asymmetries in the duration of relative periods of excitation and inhibition in the BG?**

 Asymmetries may help us infer how activity propagates through the network.

**• What cortical inputs are required to execute isometric contractions or limb displacement?**

 Are the same action-specific cells recruited during sustained contractions versus ballistic movements, but coupled to β versus γ oscillations depending on the task?

**• What is the cascade of activity changes during rapid stopping?**

 Previous research has shown that rapid stopping entails significant cortical activity in the pre-SMA and IFG (Aron et al., 2014, 2016; Chen et al., 2020; Rae et al., 2015), providing a good starting point for assessing the effects of cortical inputs on context-dependent information routing. Currently, it is unclear whether the movement-related and stop-related STN γ increase involve the same, overlapping or entirely different populations of STN cells and whether they are triggered by increased asynchronous firing or by synchronized activity. In non-human primates, a population that rapidly increased firing during action cancelation was located within the ventral part of the STN (Pasquereau and Turner, 2017). A separate population quickly decreased firing in the midst of a movement-related increase. Does the decrease result from GPe inhibition or from a sudden reduction in cortical drive?

 The GPe contains multiple cell types, two of which have distinct communication routes. (1) Prototypic cells that are more active at rest and project to all BG nuclei, including the STN, the striatum, the GPi (Abdi et al., 2015), and the TRN (Mastro and Gittis, 2015). And (2) arkypallidal cells that fire more sparsely and project exclusively to the striatum. In rodents, arkypallidal cells are strongly activated during stopping (Mallet et al., 2016) but also increase during movement (Dodson et al., 2015). Prototypic cells instead are less strongly and rapidly activated during stopping and show both movement-related increases and decreases (Dodson et al., 2015). Non-human primate recordings will be essential in revealing the functional roles of these cell types for rapid action adjustments in the primate basal ganglia.

Finally, movement inhibition may not merely be mediated by decreasing motor cortex activity but may even involve engaging parts of it, considering that motor cortex activation also seems to have a role in movement suppression (Ebbesen and Brecht, 2017).

**• What is the role of slow oscillations in proactively shaping network dynamics?**

 One possible mechanism to flexibly enable or disable a rapid response to a specific stimulus could be to proactively modulate effective connectivity between the neural ensembles that will be activated by the stimulus and the relevant action-related cortical and subcortical ensembles via temporal coupling or short-term synaptic plasticity. Some evidence for task-dependent coupling between cortical and STN activity in the β and θ range was previously shown in humans (Herz et al., 2017; Zavala et al., 2018), but reports are scarce, raising the question whether the functional relevance of these effects is still underexplored. The thalamus also appears to play an important role in goal-directed behavior (Bolkan et al., 2017; Nakajima et al., 2019) and will thus likely be relevant for understanding proactive changes in network dynamics.

**• What tasks are suitable for studying the BG’s involvement in action control?**

 If a habitual response has been established through extensive training that has created a strong direct link between a sensory stimulus and a motor response, the BG seem to be less involved (Piron et al., 2016; Klaus et al., 2019). Overtrained movements thus may be accompanied by different neural interactions compared with self-paced movements or actions requiring more sophisticated cognitive control. Another relevant observation is that the BG’s functional role in boosting movement vigor seemingly can be aided by sensory stimuli, such as loud sounds (Anzak et al., 2012). Finally, life-threatening situations seem to be yet another example triggering mechanisms compensating for BG dysfunction, resulting in “paradoxical kinesia,” where patients suddenly regain mobility when their life is at risk (Bonanni et al., 2010).

**• How do cortical inputs from associative, limbic and sensorimotor regions, interact to coordinate different behaviors? And more generally, how are intrinsic motivations and external factors integrated for online movement control? What are the mechanisms enabling BG involvement in online movement control versus learning?**

**• Is movement-related γ synchronization not only present in human but also in non-human primate CBGTC networks?**

 It is currently unclear to which extent the findings in humans translate to non-human primate recordings. Two recent studies performing spike-to-spike correlation analyses in non-human primates found no marked increase in movement-related correlations between pallidal spikes (Wongmassang et al., 2020) or spikes recorded from the GPi and the thalamus (Schwab et al., 2020). Yet, a more direct test for the presence of brief movement-related γ synchronization in non-human primates would be a spike-to-LFP phase coupling analysis, particularly during self-guided and vigorous movements.

**• What tools can be used to probe the causality of rhythmic activity?**

 Caution is warranted when interpreting electrical or optogenetic stimulation studies that often have network-wide knock-on effects (Wolff and Ölveczky, 2018). Applying stimulation without ensemble-specificity may disrupt the cross-effector channel balance that likely is key for retaining the full range of motor control functions. Broad stimulation automatically also causes synchronization, which may not be representative of physiological activation. Alternative approaches could involve optogenetic activation of sets of cells associated with distinct ensembles (Carrillo-Reid and Yuste, 2020) or neurofeedback training to prompt volitional upregulation and downregulation of oscillatory activity in a more physiological way (Khanna and Carmena, 2017; Chauvière and Singer, 2019).

## Conclusion

Based on recent findings, I described a set of hypotheses about the network interactions that may underlie flexible movement control in the human CBGTC network, hopefully serving as a starting point for further studies and further debate (see also Boxes 1, 2). I have proposed that during movement initiation, small temporal shifts of cortical activity trigger γ synchronization in the BG, kicking off the network dynamics that control movement initiation or at least regulate the movement vigor. Particularly vigorous movements seem to involve more widespread ∼70 Hz population synchrony of STN and GPe cells, causing a larger population of GPi cells to fire and pause synchronously. The idea that synchronized pauses in GPi firing may boost thalamic firing suggests that increases in STN firing could be movement-facilitatory as long as cells fire and pause synchronously, which provides a new perspective on the role of the indirect pathway in movement control.

Note that much of the evidence presented here is correlational. However, the difference in STN spike-to-cortical γ phase coupling, which was related to faster reaction times in Fischer et al. (2020), appeared already straight after the GO cue, which preceded the movement on average by half a second. Similarly, Figure 2*C* suggests that STN-GPi γ synchronization can occur 1–2 seconds before the movement. As third point, the presence of finely tuned γ activity is not only limited to movement tasks, but can also be observed when Parkinson’s patients receive clinically effective STN DBS at rest (Muthuraman et al., 2020; Wiest et al., 2021), reflecting a condition that allows them to move more easily.

Moving on to mechanism 4, I further described that during stopping of an ongoing movement, a strong cortical drive to the STN (which may also be γ-rhythmic) may shift subcortical γ-rhythmic firing. I have proposed that shifted activity could propagate to the GPi, resulting in prolonged bouts of inhibition arriving onto thalamic cells and desynchronization of thalamo-cortical γ coupling.

But what regulates the relative timing of activity for selective movement invigoration or stopping? Does the key lie in the preceding dynamics of ongoing activity or could a shift in spike timing in itself be the master command that suddenly emerges without traceable links to prior activity? What is the role of short-term synaptic plasticity? Theories about the role of prefrontal cortex in controlling working memory are rapidly evolving (Miller et al., 2018; Lundqvist et al., 2020; Sherfey et al., 2020) and will likely be key in closing the explanatory gap between movement generation and internal states.

Finally, studying β oscillations may help us understand the mechanisms underlying volitional top-down control of movement state stabilization. The intermittent nature of bursts suggests that β synchronization affects network dynamics not only for the limited duration of a burst, but potentially acts to restrict or guide how network dynamics evolve for longer periods, possibly outlasting peak synchronization for several hundreds of milliseconds.

From these hypotheses it follows that understanding cortico-BG interactions will depend not only on careful monitoring and manipulation of behaviors, but also on a detailed consideration of intrasite and intersite synchronization and resulting interactions with changes in firing rates. Moving forwards quickly will require a cross-species approach combining intraoperative recordings in patients and non-human primate studies. Already existing data could help accelerate the progress if synchronization phenomena were analyzed in more detail. Investigating directionality metrics and coupling of individual cells to LFP rhythms will hopefully help us understand what inputs drive distinct ensembles, and what input-dependent operations are performed by different BG nuclei on distinct ensembles, some of which may be movement-facilitatory or -suppressive. More detailed investigations into the synchronous nature of activity thus can provide highly valuable insights into the computations performed within the CBGTC network regardless of what causes the fluctuations in synchronous oscillations. Because of the relatively low internal complexity of the STN and the GPi, one promising approach could be to record jointly from the STN and connected sites. Computational models could then be fitted to the relationships emerging between neuronal firing, oscillations, and behavior.

Neurophysiological recording techniques have advanced such that large-scale and multi-site recordings could finally allow us to link interactions between spike patterns, synchrony and rates to understand the building blocks underlying flexible motor control, the basis of complex human behavior. Taking this approach may even allow us to improve the specificity and flexibility of neurostimulation techniques, although some neural control mechanisms may remain intractable once they go awry. The much wider implication of this approach is that fully understanding simple action control tasks may also open doors to understanding more complex cognitive functions. If a cognitive operation is probed by an immediate behavioral readout, we can work our way back from there.

## Synthesis

Reviewing Editor: Ifat Levy, Yale University School of Medicine Decisions are customarily a result of the Reviewing Editor and the peer reviewers coming together and discussing their recommendations until a consensus is reached. When revisions are invited, a fact-based synthesis statement explaining their decision and outlining what is needed to prepare a revision will be listed below. The following reviewer(s) agreed to reveal their identity: Eun Jung Hwang, Genela Morris.

The paper is a well written and thought-provoking review and opinion piece on the possible roles of gamma oscillations in the Cortico-Basal Ganglia-Thalamo-Cortical loop. The authors provide in the introduction a fairly thorough review of published work on movement related oscillations in the cortex, subthalamic nucleus and GPi. They then follow to offer four non-mutually exclusive possible mechanisms through which movement-preparation related oscillatory activity in the cortex and/or STN translates to movement invigoration, inhibition of interfering muscle activation and action cancellation. The basic idea is that spiking input from area A can have different impact on the spiking output of area B and its downstream, depending on at which phase of synchronous activity in area B the input arrives. Based on this premise, the authors built the models in which timing of the cortical spiking input relative to the underlying synchronous activity in the basal ganglia could modulate the strength of feedback signal from the basal ganglia back to the cortex, which may regulate the initiation, vigor, inhibition of movements.

The proposed mechanisms are intriguing and provide interesting topics for future study. There are, however, major issues that need to be addressed, as detailed below:

1. The literature review is comprehensive in regard to data from human recordings, but is somewhat lacking with respect to animal literature, where often reviews are cited (sometimes falsely) to support empirical claims. It would be better to refer to the original research.

Specifically:

- Lines 113-115, the sentence is misleading, please note that the cited study is a simulation.

- Similarly, line 305, ref. 92 is a computational model. Coordinated pausing of GPi neurons is not observed.

- Line 123, the refs are reviews. Addition of some original research for actual recordings of GPi neurons would be helpful.

- Line 147, not all refs on rats show movement related gamma oscillations in the CBGTC network (e.g., Leventhal et al. focus on beta oscillations, Van Der Meer et al recorded in ventral striatum, which is not classically considered to be part of the loop).

- A recent study by the Svoboda lab reports that the initiation of movements is gated by the input from the midbrain reticular and pedunculopontine nuclei to the motor cortex via thalamus (https://www.biorxiv.org/content/10.1101/2020.12.16.423127v1). That study convincingly demonstrates the causal chain of information flows that underlie the switch of the state in motor cortical activity from preparation to execution, at least in external cue driven movements. It seems highly relevant for the current study to discuss the recent paper in terms of how the proposed network model fits in or not with this new finding.

2. While the ideas expressed in the manuscript are interesting, some claims require grounding in empirical evidence or in calculation or at least they should be phrased with some reservation.

- Mechanism 1: What is the significance of the fast gamma rhythm (as opposed to slow gamma or beta) for the proposed mechanisms? E.g., are the resulting time scales more relevant for efficient synaptic integration on the recipient cells?

- Mechanism 2 relies on synchronized pauses between GPi neurons. However, non-human primate recordings found GPi cells to rarely pause, relative to the GPe, and pauses were generally not synchronized between cells (Elias et al., 2007). What evidence is there for such coordination?

- Are cells along the described axis likely to be locked to particular phases of the LFP oscillation? Specifically, is GPi firing locked to gamma oscillations, as required for mechanism 3?

- Please note that in non-human primate recordings, LFP was found to be related to cell synchrony in the cortex-Basal Ganglia in MPTP monkeys, but not under normal conditions. Since much of the human data surveyed was collected from Parkinsonian or Dystonic patients, this confound should be discussed.

3. The proposed model is inspired by many gamma synchrony-related observations in the CBGTC such as 1) strengthened gamma synchrony in the network near movement onset, 2) the modulation of gamma synchrony strength by the movement vigor, and 3) a shift of synchrony frequency in the subthalamic nucleus (STN) for the stopped movements. All these observations, including patient studies, are correlational by nature. In other words, all the observed changes in the gamma synchrony might be the consequence of the state or vigor changes rather than the causes or driving mechanisms underlying the behavioral/neuronal changes of interest. Is there strong evidence in support of the causal role of gamma synchrony? Related, even in the proposed model, the causal role of gamma synchrony is sometimes not straightforward. For instance, the first proposed mechanism for movement initiation starts with the motor cortical input being synchronized to drive gamma synchrony in the basal ganglia. If that is the case, the motor cortical activity has already changed to trigger movement execution before the basal ganglia and the gamma synchrony in the basal ganglia seems to be a consequence of such state change that has happened in the cortex, rather than driving the stage change.

4. Even if the selective movement initiation is regulated by the gamma synchrony mechanism as proposed, the model seems to be missing an important detail. To be more specific, if the timing of the spiking cortical input relative to the underlying gamma synchrony in the basal ganglia determines the selective initiation or inhibition of movements, what regulates the relative timing of the incoming input signals, which will be the ultimate master command for the selective initiation? This point is worth a discussion.

5. Figure 2: The gamma activity modulated by the movement vigor seems to follow rather than lead movement onset. Wouldn’t the gamma activity lead movement onset if the cortical input timing relative to the gamma is used to control the initiation of movements? Perhaps a significant change starts to occur before the movement onset but not obvious in the figure, in which case, it is worthwhile to note that in the figure legend.

6. Line 422: The authors proposed that at the level of the thalamus, brief barrages of potent inhibition, each arriving just before incoming excitation, could efficiently prevent ensembles from firing. Isn’t this the opposite to the proposed mechanism for the selection of the targeted movement in Figure 4C? For the selection, the potent, synchronized inhibition of the thalamus before incoming excitation was proposed as a mechanism to enable an earlier excitation of the thalamus for the incoming activity associated with the targeted movement. Please clarify these seemingly contradicting arguments for the effect of potent synchronous inhibition preceding incoming excitation.

Minor comments:

- Line 66: The authors’ description of “explorative” versus “exploitive” behaviors does not seem correct. Exploitive behavior usually refers to choosing the option with the best expected outcome, while explorative behavior refers to choosing suboptimal options against the current knowledge of the option with the best outcome.

- Line 81, correct neurosn to neurons

- Fig. 1, references style is different

- Line 254-257, the sentence is unclear.
